# Effect of mHealth on Postpartum Family Planning and Its Associated Factors Among Women in South Ethiopia: A Cluster-Randomized Controlled Trial

**DOI:** 10.3390/jcm14248703

**Published:** 2025-12-09

**Authors:** Girma Gilano, Andre Dekker, Rianne Fijten

**Affiliations:** 1 Department of Public Health Informatics, School of Public Health, College of Medicine and Health Sciences, Arba Minch University, Arba Minch P.O. Box 21, Ethiopia; 2 Department of Radiation Oncology (Maastro), GROW Institute for Oncology and Reproduction, Maastricht University Medical Centre+, 6229 ER Maastricht, The Netherlands

**Keywords:** postpartum family planning, mHealth, cluster randomized controlled trial, reproductive health, Ethiopia

## Abstract

**Introduction**: Postpartum family planning (PPFP) is a critical strategy for improving maternal and child health by preventing unintended pregnancies and optimizing birth spacing. However, PPFP uptake remains suboptimal in Ethiopia, where sociocultural barriers, limited health information, and inadequate counseling impede progress. Mobile health (mHealth) interventions have shown promise in overcoming these challenges by delivering targeted health information directly to individuals. This study aimed to evaluate the effect of an mHealth intervention on uptake and the intention to use PPFP among postpartum women in South Ethiopia. **Methods**: We conducted a cluster-randomized controlled trial in randomly selected health facilities in South Ethiopia. Pregnant women from primary hospitals and health centers were selected from registers and family folders. Data were collected using face-to-face and mobile interviews and analyzed using a generalized linear mixed model (GLMM) to account for the clustering. **Results**: The mHealth intervention significantly increased PPFP uptake (OR = 2.89, 95% CI: 1.55–5.37) and the intention to use PPFP (AOR = 2.05, 95% CI: 1.24–3.46) compared to standard care. The predicted probability of using PPFP was 85% in the intervention group. Women who discussed family planning with their partners (AOR = 2.10, 95% CI: 1.30–3.35) had a higher probability of using PPFP, and those exposed to media (AOR = 1.58, 95% CI: 1.07–2.32) had an increased likelihood of planning to use PPFP. Conversely, limited autonomy in decision-making and delays in postnatal care attendance were associated with reduced uptake and intention to use PPFP. **Conclusions**: The mHealth intervention improved uptake of PPFP and increased intention to use PPFP among postpartum women in South Ethiopia. PPFP uptake was higher in the intervention group (85%) than in the control group (68%). Partner involvement, decision-making autonomy, and media exposure emerged as significant facilitators of PPFP adoption. Scaling up mHealth interventions could address unmet family planning needs, but integration with broader strategies that address sociocultural barriers and enhanced counseling is essential. Interventions must be contextually tailored and grounded in behavioral theory (HBM, TPB, and TAM) to maximize effectiveness. Future research should examine the long-term sustainability and adaptability of mHealth approaches across diverse contexts.

## 1. Introduction

Postpartum family planning is a critical component of maternal and child health, offering significant benefits by preventing unintended pregnancies, optimizing birth spacing, and reducing maternal and infant mortality [1]. The postpartum period is a unique opportunity to address unmet family planning needs. Women frequently interact with health systems during this period. Despite this, PPFP uptake remains suboptimal in many low- and middle-income countries (LMICs) [2], including Ethiopia, where only 45.44% of postpartum women report using contraception [3,4]. The pooled prevalence of postpartum contraceptive use among women in low- and middle-income countries was 41% in 2019 [5]. The prevalence was 37.41% in the Sub-Saharan region [2].

PPFP encounters barriers in Ethiopia, such as limited access to health information, socio-cultural norms, inadequate counseling, and poor male partner involvement in family planning decisions [4,6,7,8]. These barriers are compounded by low utilization of postnatal care (PNC) services, which are pivotal for counseling on contraceptive options [4,9]. This may require an innovative strategy beyond conventional service delivery methods, including mobile health [10]. The mHealth application can provide information to oversome barriers.

Mobile health (mHealth) interventions are effective at improving health outcomes in settings with limited resources [11]. By leveraging mobile phones, mHealth can deliver targeted health information, reminders, and educational messages directly to individuals, overcoming geographical and logistical barriers [12]. Some evidence suggests that mHealth interventions have successfully improved maternal and child health outcomes, including vaccination uptake [13], antenatal care attendance [14], postpartum family planning [15], and breastfeeding [16]. However, limited research has explored their impact on postpartum contraceptive use and the intention to adopt family planning.

Despite some efforts to enhance postpartum family planning in Africa, the problem remains prevalent. Ethiopia lacks sufficient evidence to support the expansion of mHealth. This study aims to evaluate the effect of a mHealth intervention on uptake (primary outcome) and the intention to use PPFP (secondary outcome) among postpartum women in South Ethiopia. The intervention involved delivering health information and reminder messages via mobile phones to women in the intervention group, while the control group received standard care. The findings of this trial can contribute to growing the potential of mHealth to address unmet family planning needs in LMICs and provide actionable recommendations for scaling up mHealth interventions. South Ethiopia was selected by convenience due to proximity. Given the nature of health status and cultural background, the findings may be generalizable to all regions of Ethiopia and to comparable contexts throughout Africa.

## 2. Methods and Materials

### 2.1. Study Design and Setting

A cluster-randomized controlled trial (cRCT) was employed to assess the effect of an SMS-based mHealth intervention on key maternal and child health outcomes, including postpartum family planning uptake, childhood vaccination, prelacteal feeding, and exclusive breastfeeding. Health facilities (primary hospitals, health centers, and health center-associated health posts) were randomly assigned to either the intervention group, which received structured SMS text messages and reminders, or the control group, which received standard care.

This study specifically focused on evaluating the impact of mHealth interventions on the uptake and the intention to use PPFP among postpartum women. The study was conducted from 1 May 2024 to 31 January 2025, with data collection facilitated by trained professionals under the supervision of experienced researchers. Participants were recruited from March to April 2024.

The study examines various outcomes, including postpartum family planning, exclusive breastfeeding, prelacteal feeding, and childhood vaccinations, presented in separate reports. Follow-up began during ANC and continued for up to six months postpartum, so the duration of the outcomes may vary slightly. The data collection for this outcome commenced on 31 January 2025.

### 2.2. Participants

Initially, we randomly selected 20 Health Facilities (HFs) from the available 102 from an opaque envelope, followed by individual women from ANC registers (health centers and district hospitals) and family folders (health posts) using simple random sampling. This study outcome involved several months of ANC and PNC follow-up to evaluate the effect of mHealth on mothers’ PPFP. Health professionals were blinded to group allocation (intervention vs. control) and to eligibility status and final enrolment. No minority participant mothers were identified in the sample. All included mothers can read, understand, and write Amharic.

The inclusion and exclusion criteria were:

Pregnant women aged 18 years or older were eligible for enrolment, with a gestational age of 16 to 28 weeks at recruitment. Recruitment took two months, and the minimum gestational age at the start of the intervention was 24 weeks. Participants were proficient in reading and writing and capable of independently managing mobile phones. Participation excluded those planning to relocate before the end of the follow-up, mothers experiencing high-risk pregnancies requiring specialized care, and women who reported mobile network internet interruptions in their area during the pre-intervention interview. Network interruptions occur when services stop or malfunction for hours. These outages are often caused by power interruptions, particularly at telecom stations that lack backup power sources such as generators. Participants were excluded if they had significant health side effects or known FP-related complications that could affect their participation. During the study recruitment, no woman reported these exclusions.

### 2.3. Sample Size Determination

The required sample size was calculated using data from a prior mHealth intervention study targeting child vaccination outcomes. The vaccination prevalence was 70.9% among controls and 82.6% in the intervention group [17]. Assuming a minimum detectable difference of 12% in PPFP prevalence, an intra-cluster correlation coefficient (ICC) of 0.011 [18], a power of 80%, and a margin of error of 5%, the total required sample size becomes 680 mothers across 20 health facilities, with an average of 34 participants per HF. During recruitment, 680 women met eligibility criteria and were enrolled. We used Equal allocation to two groups (340 in the intervention group and 340 in the control group). Participants were recruited across 10 health facilities, yielding 34 participants per facility (340 per group).

A total of 672 participants were required to achieve 80% power to detect a 12 percentage-point difference in vaccinated children between groups at α = 0.05 (two-sided). During recruitment, 680 participants were enrolled to ensure adequate power in the face of potential loss to follow-up and data quality issues; all randomized participants were included in the primary analysis.

### 2.4. Randomization and Allocation

Two-stage randomizations were carried out. In the first stage, health facilities (HFs) were selected, and in the second stage, individual participants were chosen. Among 102 eligible health facilities (HFs) in the zones, 20 clusters (10 intervention and 10 control) were randomly selected from sealed opaque envelopes containing sequential numbers and HF names. Two individuals outside the study team independently assigned clusters to intervention or control by drawing from the same envelope. Participants from each health facility (HF) and its catchment area were recruited using simple random sampling. ANC registration numbers and family folder numbers served as sampling frames for the recruitment team. Randomization of clusters ensured no overlap in participant allocation (intervention and control groups). The research technical assistant prepared the sealed envelopes for HFs, and the recruitment team enrolled individual participants.

### 2.5. Intervention

The intervention group received postpartum family planning information and reminders via a mobile phone–based mHealth system. Health messages were developed by the principal investigator, tested in non-participating clusters, and uploaded to FrontlineSMS by the trial manager. The messages were informed by the Theory of Planned Behavior and framed [19] in relation to prior literature [20,21,22], RMNCH/FP national strategies, behavioral change communication frameworks, and WHO guidelines [23]. The knowledge and motivation pathway describes how health information delivery, education, reminders, and decision support shape maternal and caregiver knowledge, attitudes, self-efficacy, and intentions to use health services and to practice healthier behaviors. This pathway integrates several theories, such as the Health Belief Model (HBM) and the Theory of Planned Behavior (TPB) (linking beliefs and perceived barriers to action) [19,24].

The Technology Acceptance Model (TAM) [25] explains perceived usefulness and ease of use, with extensions to include social influence, facilitating conditions, and user experience. The Integrated Behavior Model (IBM) extends the Theory of Reasoned Action (TRA) and TPB, building on Fishbein’s work to connect beliefs to behavior. The Social-Ecological Model (SEM) is a framework that explains how behavior and health outcomes are shaped by multiple levels: individual, interpersonal, community, organizational, and societal factors [26]. It consolidates constructs from these earlier models, such as attitudes, perceived norms, and personal agency, while adding behavioral skills and environmental constraints to better predict and explain human behavior, especially in health contexts [27]. The behavioral model is used only to guide message design and assessment metrics. Behavioral constructs (cue to action, feedback/reinforcement, and social norming) are embedded in message delivery, but no dynamic tailoring or algorithmic adaptation beyond these principles was applied. Engagement metrics (message opening rates), behavioral outcomes (self-reported adherence), and psychosocial mediators (self-efficacy, perceived barriers) were assessed at the end of each objective. The trial manager programmed, scheduled, and monitored message delivery and read-status reports. During the postpartum period, a total of 6760 messages were sent with a 98.5% delivery success rate. Messages were transmitted securely via a VPN provided by the Ethiopian Telecommunication Corporation and hosted in the Health Informatics Department at Arba Minch University.

**Figure 1:**
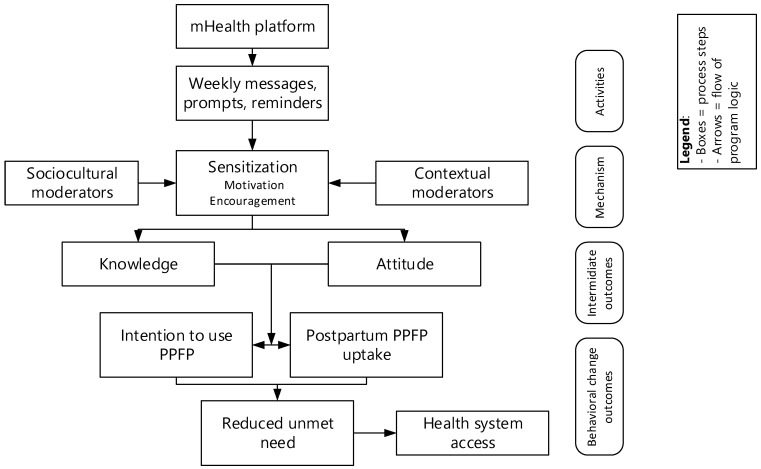
A visual theory diagram showing the flow and mechanism of mHealth impact.

Participants in the intervention arm received tailored, time-specific SMS messages. The messages focus on ANC and PNC visit reminders, postpartum family planning counseling and appointment reminders, vaccination schedules (e.g., BCG, Penta1–Penta3), encouragement of exclusive breastfeeding, nutrition education, danger-sign awareness, and partner involvement and family support. The messages were designed to be ≤160 characters, in the local language, culturally appropriate, and action-oriented, sent once per week (one week of health information followed by a reminder in the next week), and delivered via a secure, automated mHealth platform (Figure 1).

Sample SMS Messages:

“Remember your appointment with the health professional at your follow-up health facility for this week. “Exclusive breastfeeding for 6 months is best. No water or other foods needed.” “Use birth control drugs, plan your next pregnancy.” “Vaccination protects against diseases and allows children to thrive.”

### 2.6. Intervention Monitoring and Quality Assurance

The trial manager monitored SMS delivery using system logs. For any non-delivery, opt-out, or unread messages, the reason was investigated, and a phone call was made to confirm whether the participant remained in the trial. If the mother could not be reached at any of the addresses provided during registration, she was recorded as lost to follow-up. Protocol fidelity was maintained through weekly supervision. A midline data review was conducted to adjust timing or content if needed. Reading or message engagement was assessed three times (every three months) with a sample of 100 mothers (self-report).

Control Group: Participants in the control group received standard maternal and child health services and counseling routinely provided by health facilities, without mobile phone-based messaging.

### 2.7. Data Collection

Data were collected through in-person and telephone interviews conducted by trained professionals blinded to group allocation and supervised to ensure quality. A structured questionnaire captured sociodemographic characteristics, reproductive health factors, family planning factors, and health information and media exposure to assess their influence on postpartum family planning (PPFP). Supervisors ensured consistency and data quality throughout the collection process. The questionnaire was adapted from prior studies on mHealth applications and tested in 5% of non-selected clusters to assess validity [28,29,30,31]. The study compared mHealth effects between control and intervention groups using self-reported data and did not include a comprehensive document review.

### 2.8. Variables

Sociodemographic Characteristics: Residence, maternal and partner education, family size, religion, maternal occupation, and partner occupation.

Reproductive Health Factors: Age of the youngest child, number of children, frequency of ANC visits, and time to first postnatal care (PNC).

Family Planning Factors: Current use of PPFP, previous use of family planning (ever used FP), intention to use PPFP, discussing family planning with a partner, husband’s approval of family planning, and decision-making autonomy.

Health Information and Media: The exposure to maternal and child health (MCH) information and access to media (e.g., television and radio).

### 2.9. Primary and Secondary Outcomes

Primary Outcome: The effect of mHealth on self-reported use of postpartum family planning, which is measured as a binary outcome (yes/no). PPFP can be any available modern FP methods that can include injectables, oral contraceptive pills, and implantable methods.

Secondary Outcome: Intention to use PPFP, also measured as a binary outcome (yes/no). Mothers may intend to use any of the modern birth control methods available in the postpartum period.

### 2.10. Statistical Analysis

Statistical analyses accounted for the clustered nature of the data using generalized linear mixed models (GLMMs) with random intercepts to account for between-cluster variability. Generalized Linear Mixed Models are powerful statistical models used to analyze data where the outcome variable may not be normally distributed, whether it is binary, count, or categorical data. The data have a hierarchical or clustered structure (participants nested in health facilities, or individuals in communities).

The model is mathematically expressed as:

$$g\left(\mu ij\right)=\;\beta 0+\beta 1X1+\beta 2X2+....+uj$$

where

- $g\left(\cdot \right):\mathrm{Link}\;\mathrm{function}\;(\mathrm{logit})$.
- μij: Expected value of the outcome for individual iii in cluster j.
-
β0, β1, β2: Fixed−effect coefficients.

-
uj: Random effect for cluster j.

**Figure 2:**
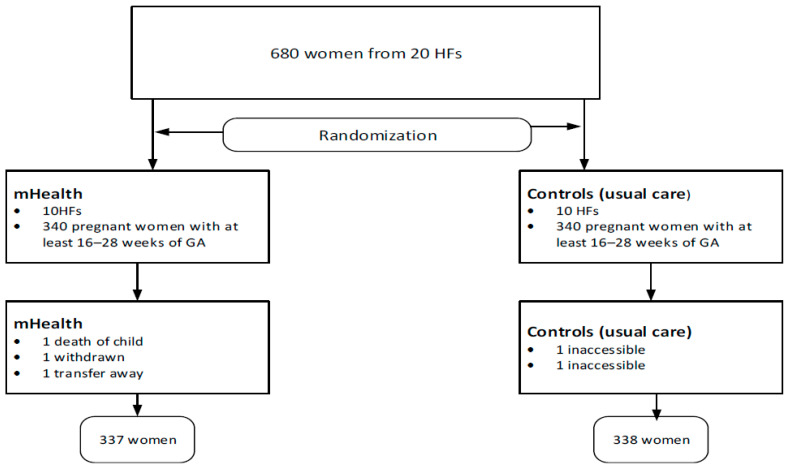
A flow chart depicting the research process.

GLMMs extend generalized linear models (GLMs), such as logistic regression, by incorporating random effects to account for correlations within groups or clusters. Because the outcome is binary (yes/no), we used a logit link. Fixed effects represent the main variables of interest (e.g., age, education, occupation, family planning) and estimate effects across all subjects. In generalized linear mixed models, the link function maps the expected value of the dependent variable (the mean of the outcome) to the linear predictor, which comprises both fixed and random effects. The logit link transforms a probability (bounded between 0 and 1) into a continuous scale from negative infinity (−∞) to positive infinity (+∞). This approach permits the probability of the outcome to be modeled linearly as a function of the predictors. Random effects capture between-cluster variation (e.g., health center, residence) and allow the intercept (and optionally slopes) to vary by group, thereby accounting for intra-cluster correlation (ICC). The intervention’s effectiveness is reported as odds ratios (ORs) with 95% confidence intervals (CIs). ICCs were estimated to assess within-cluster homogeneity. Model fit was evaluated using the Akaike Information Criterion (AIC) and Bayesian Information Criterion (BIC). Analyses were conducted in Stata version 14. Figure 2 summarizes the research process. Pregnant women (680) from 20 health facilities, with gestational age of 16–28 weeks, were recruited into control and intervention arms. Four participants were lost and did not start the intervention. One participant was lost to follow-up after commencing the intervention. One of the mothers reported loss of the baby to respiratory illness in the second month (Figure 2 and Supplementary File S1).

## 3. Results

Of the 676 women followed in the postpartum period, 675 were available at the end of the follow-up. The groups had equal sizes and showed no significant baseline differences in FP counseling during ANC (*p* = 0.6, df = 0.21) or in prior FP use history (*p* = 0.8, df = 0.05). However, the discussion of FP with a partner, the plan to use PPFP, and the current use of PPFP showed significant differences after the intervention (Table 1).

Women in the intervention had a higher probability of using PPFP than those in the control (AOR = 2.89, 95% CI: 1.55–5.37; *p* < 0.001). This shows that the intervention significantly increased the likelihood of PPFP uptake, demonstrating the importance of mobile phone-based health information and reminders. Women whose partners belonged to job categories other than farmer, government employee, merchant, or self-employed had a 67% lower probability of using postpartum family planning (PPFP) (AOR = 0.33, 95% CI: 0.12–0.96; *p* < 0.05). Self-employed women had a 76% reduction in the likelihood of PPFP use (AOR = 0.24, 95% CI: 0.07–1.23; *p* < 0.05). Women who discussed family planning with their partners were more likely to use PPFP, with a twofold increase in probability (AOR = 2.10, 95% CI: 1.30–3.35; *p* < 0.01). Finally, when the husband is the primary decision maker on family planning, the probability of using PPFP is reduced by 67% (AOR = 0.33, 95% CI: 0.21–1.71, *p* < 0.001) (Table 2).

The predicted probability of using PPFP was 85% in the intervention group and 68% in the control group. The residual is 0.001 with a Standard Deviation (SD) of 0.38, nearly 0, indicating a perfect match or prediction.

### Plan to Use PPFP (Secondary Outcome)

Women in the intervention group had a higher likelihood of planning to use PPFP than those in the control group (AOR = 2.05, 95% CI: 1.24–3.46; *p* < 0.05). This shows that mHealth intervention increased women’s intention to use PPFP, demonstrating the importance of health information and reminder messages delivered via mobile phones. Women whose husbands approved of family planning had more than 3 times the probability of planning to use PPFP (AOR = 3.41, 95% CI: 2.25–5.15; *p* < 0.001). Women who discussed family planning with their partners had a 55% reduced likelihood of planning to use PPFP (AOR = 0.45, 95% CI: 0.29–0.68; *p* < 0.001). Women who accessed media (e.g., radio, TV, or other platforms) had double the chance of planning to use PPFP (AOR = 1.58, 95% CI: 1.07–2.32; *p* < 0.05) (Table 3).

## 4. Discussion

Through a cluster-randomized controlled trial, we evaluated the effect of mHealth interventions on postpartum family planning (PPFP) uptake and the intention to use PPFP among women in South Ethiopia. Women in the intervention group had a higher probability of using PPFP than the control group. This aligns with evidence from previous studies that demonstrated the effectiveness of mHealth interventions in improving contraceptive uptake and reproductive health outcomes [8]. Another study in North Ethiopia showed PPFP increased by 13% compared to our 17% [7]. Although North Ethiopia was a highly war-affected area, mHealth demonstrated higher achievement. The comparative evidence originates from a body of cumulative randomized controlled trials conducted in developing countries, enabling cross-study comparability. The findings highlight the potential of mHealth in enhancing reproductive health behaviors, particularly in resource-limited settings such as Ethiopia. Delivering health information and reminders via mobile phones might increase awareness and address common misconceptions about PPFP.

Partners’ non-standard occupations within the country (e.g., farming, government employee, merchant, or self-employed) were associated with reduced PPFP uptake among women. The direction and magnitude of this association are consistent with prior literature [32]. The observed negative association between partners’ non-standard occupations and postpartum family planning uptake likely reflects socioeconomic and structural disparities. Non-standard employment is often linked to income instability, limited exposure to health information, and weaker access to health facilities. Moreover, men in informal occupations may hold more traditional gender norms, constraining women’s decision-making autonomy regarding contraception. These mechanisms align with the social determinants of health framework, which posits that occupational status shapes both access to services and reproductive agency [33,34].

The combination of limited education and unstable or informal employment among partners (e.g., daily laborers) can lower the quality of life and constrain decision-making processes relevant to PPFP. Economic conditions may modulate access to information, autonomy, and resources that support PPFP uptake. Therefore, family capacity-building initiatives could be crucial for achieving PPFP objectives. The literature also suggests that self-employed women are at higher risk of PPFP underuse, whereas employed women tend to use PPFP more frequently [32,35]. Enhancing women’s job opportunities and educational attainment can improve access to health information, thereby increasing birth-spacing autonomy and expanding knowledge relevant to PPFP.

Maternal autonomy and partner involvement influenced PPFP uptake. Women who discussed family planning with their partners were more likely to adopt PPFP, consistent with studies that emphasize the role of male partner engagement in reproductive health decisions [36]. Conversely, women whose husbands were the sole decision makers had a reduced success rate of using PPFP. The finding is consistent with evidence from other studies [37,38]. These findings underscore the need for targeted interventions to promote gender-equitable decision-making in family planning.

The intervention also strengthened women’s intention to use PPFP, with those in the intervention group demonstrating more intention to adopt PPFP. Evidence from low- and middle-income countries shows that mHealth enhances the use and intention to use PPFP [39,40]. According to the theory of planned behavior, intention is the most immediate determinant of behavior, but not all intentions translate into action. Barriers such as access to services, partner opposition, cultural norms, or postpartum complications often prevent intended use from becoming actual use [19]. Thus, actual PPFP use reflects a realized behavioral outcome, while intention reflects only potential readiness. This finding suggests that mHealth interventions may enhance women’s intention to use postpartum family planning (PPFP). Moreover, partner communication emerged as the strongest predictor: women who discussed family planning with their partners were significantly more likely to plan for PPFP. The evidence is consistent with other studies showing that integrating husband involvement into FP is worth consideration during implementation [2,38,41]. When the husband decides regarding PPFP, women show a decreased likelihood of planning for PPFP. Evidence suggests that women often avoid using PPFP due to the fear of their husbands’ disapproval, leading to unnecessarily limiting their use [42]. Thus, the promotion of postpartum family planning (PPFP) should center on empowering women, while ensuring context-specific approaches and cultural sensitivity.

Media exposure also influenced intentions to use PPEP, echoing findings from similar studies in LMICs that highlighted the power of mass communication in driving health behaviors [43,44,45]. Overall, the intervention in this study was guided by behavioral models, which facilitated the development of contextualized and culturally tailored messages to promote behavior change.

The integration of mobile health (mHealth) within Ethiopia’s health system is contingent on multiple factors, including existing infrastructure, data transmission reliability, and the application of behavioral frameworks that enhance cost-effectiveness. The intervention leveraged Ethio-Telecom for national connectivity and was hosted on university servers, thereby reducing capital costs. This arrangement supported high SMS delivery success and low marginal costs per user, indicating strong reliability with minimal data loss. The design incorporated behavioral change models to promote adoption without requiring extensive personalization or field-level interventions. Additionally, the approach has the potential to substitute certain face-to-face counseling activities, potentially reducing the demand for health extension workers (HEWs) and clinic visits, as well as transportation costs. Evidence [38,39] shows that mHealth interventions in similar contexts improved contraceptive uptake with minimal incremental cost [15,39,40]. Some studies estimate the cost of family planning to be approximately $8.16 per woman per year (based on 2020 US $8160 per 1000 women). Family planning costs in low- and middle-income countries range from $10–20 per user, with potential reductions to $2–12 per person per year when mHealth is applied [46,47].

## 5. Strengths and Limitations

The cluster randomized controlled trial enhances the validity and generalizability of the findings. Random effects addressed clustering and accounted for intraclass correlation, ensuring robust statistical inference. Assessing both actual PPFP use and the intention to use PPFP provides comprehensive insights into reproductive health behaviors. The application of different behavioral conceptual models to obtain insights into cultures, contexts, and the nature of the problems enabled the successful delivery and acceptance of mHealth messages.

While the mHealth interventions in this study demonstrated strong efficacy, several implementation barriers, including infrastructural constraints, human resource limitations, sociocultural dynamics, system integration challenges, and sustainability concerns, were not captured in the measured outcomes. Addressing these barriers through implementation science, partner engagement, and policy integration is essential for achieving real-world, long-term impact of mHealth on maternal and child health in Ethiopia and similar settings. Additionally, the results section shows partner involvement in PPFP decision-making. Not including a partner in the design can be considered a certain limitation of the study, as partners indirectly influenced decisions.

Reliance on self-reported PPFP use and planning may introduce recall or social desirability bias. Factors such as distance to health facilities or sociocultural norms were not explicitly measured but could influence PPFP uptake and intentions. The study did not assess the long-term sustainability of mHealth impacts, limiting insights into continued PPFP use beyond the study period. The protocol’s early publication led to modifications in the methodologies beyond publication. This may cause some confusion that should be considered accordingly. This design creates barriers for non-literate users and those without access to mobile phones, biasing benefits toward wealthier, more educated groups. The authors acknowledge follow-up misses or discontinuation, behavioral theory mediation not evaluated, and the Hawthorne effect (mothers may change their behavior and intentionally report use of the service).

## 6. Conclusions

This study demonstrates that mHealth interventions can improve uptake and the intention to use PPFP among postpartum women in South Ethiopia. Partner communication, media exposure, and maternal autonomy emerged as critical determinants of reproductive health behaviors. While mHealth offers a promising approach to enhancing family planning uptake, integrating it with broader strategies to address sociocultural barriers is essential for sustained impact. The success of mHealth was driven by the behavioral change frameworks. Maternal decision-making on contraception is understood as a function of perceived need, perceived barriers, and external cues to action (HBM). Regular mobile reminders served as these “cues to action,” reinforcing knowledge, addressing misconceptions, and sustaining motivation to adopt family planning after delivery.

Policy Recommendations:

Government policies should integrate mHealth into national reproductive health strategies, guaranteeing funding and infrastructure for broad adoption. Collaborating with media to disseminate precise, engaging family planning information can significantly enhance community awareness.

Further Research:

Future studies can explore the long-term effects of mHealth interventions on sustained contraceptive use. Investigating the negative association between maternal occupation and PPFP could uncover potential conflicts or gaps in the provision of health messages. Research on the impact of mHealth interventions in diverse sociocultural contexts will help acclimate strategies for broader applicability.

Implications

The positive effects of mHealth on PPFP uptake and intentions suggest that similar interventions can be scaled to other regions with low contraceptive prevalence. Government and non-governmental organizations (NGOs) can prioritize mHealth initiatives in their reproductive health programs, focusing on culturally sensitive messaging.

Training healthcare providers to deliver consistent, integrated messages on maternal health and family planning is crucial. Additionally, health facilities should incorporate partner-inclusive counseling sessions to promote shared decision-making in family planning. Empowering women to make the final decision regarding PPFP could enhance uptake of postpartum family planning. Scaling the mHealth intervention requires strengthening digital infrastructure by ensuring reliable mobile network coverage, establishing secure SMS platforms integrated into the national HMIS, and equipping health facilities with functional computers and power backup systems. Health workers must receive practical training in digital literacy, system troubleshooting, message monitoring, and data privacy protocols to support routine implementation. Sustainable adoption further requires dedicated funding streams, including telecom agreements, software maintenance budgets, and capacity-building funds, secured through government allocation or donor partnerships to ensure long-term viability beyond pilot phases.

Partner-counseling sessions can be structured as short, integrated modules delivered during ANC or early postnatal visits, beginning with a brief joint orientation on key maternal and child health topics (EBF, pre-lacteal avoidance, PPFP, vaccination), followed by a guided dialogue where partners discuss shared roles, decision-making, and support needs. Each session should end with action planning, where couples identify concrete tasks, such as reminders for vaccination visits, support for breastfeeding, or joint decisions on postpartum family planning, and receive synchronized mHealth messages reinforcing counseling content at home.

To ensure equity for women who lack phones or have low literacy, the mHealth program should pair digital content with community-based delivery methods, such as health extension worker home visits and women’s group sessions to reflect on digital content. Voice messages, interactive voice response (IVR), and partner-linked phone enrolment can facilitate access for women without personal devices. Additionally, using pictorial aids and oral counseling will help low-literacy women effectively receive essential maternal and child health information.

The optimal timing for postpartum mHealth messages is around 7:00 P.M., when most mothers have completed daytime tasks and are more available to read or listen to messages, aligning with engagement patterns observed in the study. Messages that are not delivered or opened should be resent within 24 h, preferably the following morning between 8:00 and 10:00 A.M., to maximize reach while avoiding alert fatigue. This two-time-window strategy balances caregiver workload, salience, and convenience during the postpartum period during scaling up.

## Figures and Tables

**Table 1:** The distribution of baseline and post-intervention FP characteristics.

Variables	Categories	Control	Intervention	X^2^
		Number (%)	Number (%)	Df (*p*-Value)
Ever heard PPFP	No	170 (50.30)	207 (61.24)	7.82 (0.004)
	Yes	168 (49.70)	131 (38.76)	
Ever used FP	No	216 (63.91)	212 (62.72)	0.05 (0.8)
	Yes	122 (36.09)	126 (37.28)	
Using PPFP	No	106 (31.36)	51 (15.09)	24.67 (0.001)
	Yes	232 (68.64)	287 (84.91)	
Plan to use PPFP	No	218 (64.50)	160 (47.48)	20.12 (0.001)
	Yes	120 (35.50)	177 (52.52)	
Discuss FP with the partner	No	189 (55.92)	113 (33.53)	33.75 (0.001)
	Yes	149 (44.08)	224 (66.47)	
Husband approves FP	No	167 (49.41)	141 (41.84)	3.75 (0.05)
	Yes	171 (50.59)	196 (58.16)	
Decision maker on the FP issue	Mine	152 (44.97)	171 (50.74)	6.50 (0.04)
	Husband’s	160 (47.34)	154 (45.70)	
	Discussion	26 (7.69)	12 (3.56)	
FP counseling during ANC	No	280 (82.84)	282 (83.43)	0.04 (0.84)
	Yes	58 (17.16)	56 (16.57)	

**Table 2:** The fixed-effect output of mHealth on postpartum FP and associated factors.

Variables	Categories	AOR (95% CI)
Treatment group	Controls	1
	Intervention	2.89 (1.55–5.37) ***
Partner occupation	Farmer	1
	Government employee	0.92 (0.49–1.70)
	Merchant	0.75 (0.43–02.34)
	Self-employee	0.34 (0.28–1.89)
	Others	0.33 (0.12–0.96) *
Maternal occupation	Farmer	1
	Housewife	1.28 (1.27–2.12)
	Government employ	1.08 (1.70–2.04)
	Merchant	1.46 (1.55–3.32)
	Self-employ	0.24 (0.07–1.23) *
Discuss FP with the partner.	No	1
	Yes	2.10 (1.30–3.35) **
Decision maker on FP	Mine	1
	Husband’s	0.33 (0.21–1.71) ***
	Discussion	0.53 (0.22–1.28)
Model evaluation		
Criteria	Random intercept model	Fixed effect model
Var(_cons)	0.21	0.15
ICC	0.06	0.04
AIC	705	681
BIC	719	802

Key: * = *p* < 0.05, ** = *p* < 0.01, & *** = *p* < 0.001. NB: Only significant coefficients were converted to AOR.

**Table 3:** The fixed-effect output of mHealth on the plan to use PPFP and associated factors.

Variables	Categories	AOR (95% CI)
Treatment group	Controls	1
	Intervention	2.05 (1.24–3.46) **
Husband approves FP	No	1
	Yes	3.41 (2.25–5.15) ***
Decision maker on family planning	Mine	1
	Husband’s	0.45 (0.29—0.68) ***
	Discussion	0.32 (0.13–1.24)
Use of media	No	1
	Yes	1.58 (1.07–2.32) *
Model evaluation		
Criteria	Random intercept model	Fixed effect model
Var(_cons)	0.31	0.17
ICC	0.09	0.05
AIC	892.33	720
BIC	905.88	755

Key: * = *p* < 0.05, ** = *p* < 0.01, and *** = *p* < 0.001. ALO: adjusted log odds.

## Data Availability

We will make the data generated during this study fully available upon proper request.

## Supplementary Materials

The following supporting information can be downloaded at: https://www.mdpi.com/article/10.3390/jcm14248703/s1, File S1: mHealth SMS System Implementation and Monitoring Framework.

## Abbreviations

PPFP: Postpartum Family Planning; FP: Family Planning; HFs: Health Facilities; UNICEF: United Nations Children Emergency Fund; ANC: Antenatal Care; PNC: Postnatal Care; ICC: Intra-Cluster Correlation; HEWs: Health Extension Workers; AIC: Akaike Information Criterion; BIC: Bayesian Information Criterion; GA: Gestational Age; ODK: Open Data Kit; and AOR: Adjusted Odds Ratio; RMNCH: Reproductive, Maternal, Newborn and Child Health.

## References

[1] WHO (2013). Programming Strategies for Postpartum Family Planning [Internet]. https://iris.who.int/bitstream.

[2] Yemane T.T., Bogale G.G., Egata G., Tefera T.K. (2021). Postpartum Family Planning Use and Its Determinants among Women of the Reproductive Age Group in Low-Income Countries of Sub-Saharan Africa: A Systematic Review and Meta-Analysis. Int. J. Reprod. Med..

[3] Central Statistical Agency (CSA) [Ethiopia] and ICF Ethiopia Demographic and Health Survey 2016. Addis Ababa, Ethiopia, and Rockville, Maryland, USA: CSA and ICF. https://www.dhsprogram.com/pubs/pdf/FR328/FR328.pdf.

[4] Tesfu A., Beyene F., Sendeku F., Wudineh K., Azeze G. (2022). Uptake of postpartum modern family planning and its associated factors among postpartum women in Ethiopia: A systematic review and meta-analysis. Heliyon.

[5] Dev R., Kohler P., Feder M., Unger J.A., Woods N.F., Drake A.L. (2019). A systematic review and meta-analysis of postpartum contraceptive use among women in low- and middle-income countries. Reprod. Health.

[6] Tilahun T., Bekuma T.T., Getachew M., Oljira R., Seme A. (2022). Barriers and determinants of postpartum family planning uptake among postpartum women in Western Ethiopia: A facility-based cross-sectional study. Arch. Public Health.

[7] Cherie N., Wordofa M.A., Debelew G.T. (2024). Effectiveness of an Interactive Mobile Health Intervention (IMHI) to enhance the adoption of modern contraceptive methods during the early postpartum period among women in Northeast Ethiopia: A cluster Randomized Controlled Trial (RCT). PLoS ONE.

[8] Perinpanathan T., Maiya S., van Velthoven M.H.H., Nguyen A.T., Free C., Smith C. (2023). Mobile phone-based interventions for improving contraception use. Cochrane Database Syst. Rev..

[9] Meskele M., Sadamo F.E., Angore B.N., Dake S.K., Mekonnen W., Kebede A.T., Adinew Y.M., Shikur B., Assegid M., Firdu N. (2024). Barriers and enablers to the implementation of immediate postpartum and post-abortion family planning service integration in primary health care units of Wolaita Zone, Southern Ethiopia: A baseline study for implementation research. PLoS ONE.

[10] Sun S., Simonsson O., McGarvey S., Torous J., Goldberg S.B. (2024). Mobile phone interventions to improve health outcomes among patients with chronic diseases: An umbrella review and evidence synthesis from 34 meta-analyses. Lancet Digit. Health.

[11] Tumuhimbise W., Theuring S., Kaggwa F., Atukunda E.C., Rubaihayo J., Atwine D., Sekandi J.N., Musiimenta A. (2024). Enhancing the implementation and integration of mHealth interventions in resource-limited settings: A scoping review. Implement. Sci..

[12] Aung B., Mitchell J.W., Braun K.L. (2020). Effectiveness of mHealth interventions for improving contraceptive use in low-And middle-income countries: A systematic review. Glob. Health Sci. Pract..

[13] Mekonnen Z.A., Gelaye K.A., Were M.C., Gashu K.D. (2019). Effect of mobile text message reminders on routine childhood vaccination: A systematic review and meta-analysis. Syst. Rev..

[14] Rahman M., Yamaji N., Nagamatsu Y., Ota E. (2022). Effects of mHealth Interventions on Improving Antenatal Care Visits and Skilled Delivery Care in Low- and Middle-Income Countries: Systematic Review and Meta-analysis. J. Med. Internet Res..

[15] Harrington E.K., Drake A.L., Matemo D., Ronen K., Osoti A.O., John-Stewart G., Kinuthia J., Unger J.A. (2019). An mHealth SMS intervention on Postpartum Contraceptive Use among Women and Couples in Kenya: A randomized controlled trial. Am. J. Public Health.

[16] Qian J., Wu T., Lv M., Fang Z., Chen M., Zeng Z., Jiang S., Chen W., Zhang J. (2021). The Value of Mobile Health in Improving Breastfeeding Outcomes Among Perinatal or Postpartum Women: Systematic Review and Meta-analysis of Randomized Controlled Trials. JMIR mHealth uHealth.

[17] Mekonnen Z.A., Gelaye K.A., Were M., Tilahun B. (2021). Effect of mobile phone text message reminders on the completion and timely receipt of routine childhood vaccinations: Superiority randomized controlled trial in Northwest Ethiopia. JMIR mHealth uHealth.

[18] Taljaard M., Donner A., Villar J., Wojdyla D., Velazco A., Bataglia V., Faundes A., Langer A., Narváez A., Valladares E. (2008). Intracluster correlation coefficients from the 2005 WHO Global Survey on Maternal and Perinatal Health: Implications for implementation research. Paediatr. Perinat. Epidemiol..

[19] Ajzen I. (1991). The theory of planned behavior. Organ. Behav. Hum. Decis. Process..

[20] Gilano G., Sako S., Dileba T., Dekker A., Fijten R. (2023). Assessing the effect of mHealth on child feeding practice in African countries: Systematic and meta-analysis. J. Health Popul. Nutr..

[21] Gilano G., Sako S., Molla B., Dekker A., Fijten R. (2024). The effect of mHealth on childhood vaccination in Africa: A systematic review and meta-analysis. PLoS ONE.

[22] Gilano G., Zeleke E.A., Dekker A., Fijten R. (2024). Contextual success and pitfalls of mHealth service for maternal and child health in Africa: An Intervention, Context, Actors, Mechanism, and Outcome (ICAMO) framework guided systematic review of qualitative evidence. BMC Pregnancy Childbirth.

[23] Wagle K. Behavior Change Communication (BCC): Importance and Strategies. 23 September 2019; pp. 1–8. https://publichealthnotes.com.

[24] Rosenstock I.M. (1974). Historical origins of the health belief model. Health Educ. Monogr..

[25] Davis F.D., Al-Suqri M.N., Al-Aufi A.S. (1989). Technology Acceptance Model: TAM. Information Seeking Behavior and Technology Adoption.

[26] Bronfenbrenner U. (1979). The Ecology of Human Development: Experiments by Nature and Design.

[27] Fishbein M., Yzer M.C. (2003). Using theory to design effective health behavior interventions. Commun. Theory.

[28] Megersa N.D., Tariku E.Z., Yesera G.E., Gutema B.T. (2021). Prevalence of prelacteal feeding and its associated factors among mothers of under-24-month-old children at arba minch zuria district, Ethiopia: A cross-sectional study. SAJCH S. Afr. J. Child Health.

[29] Downs S.M., Gueye D., Sall M., Ndoye B., Sarr N.N., Sarr M., Mboup S., Alam N.A., Diouf A., Merchant E.V. (2023). The impact and implementation of an mHealth intervention to improve infant and young child feeding in Senegal: IIMAANJE protocol for a cluster randomized control trial. Front. Public Health.

[30] Gilano G., Dekker A., Fijten R. (2024). Effect of mobile phone messaging on uptake of maternal and child health service in southern Ethiopia: Protocol for cluster randomized controlled trial. Clin. Nutr. Open Sci..

[31] Adam M., Tomlinson M., Le Roux I., Lefevre A.E., McMahon S.A., Johnston J., Kirton A., Mbewu N., Strydom S.L., Prober C. (2019). The Philani MOVIE study: A cluster-randomized controlled trial of a mobile video entertainment-education intervention to promote exclusive breastfeeding in South Africa. BMC Health Serv. Res..

[32] Feriani P., Yunitasari E., Efendi F., Krisnana I., Ernawati R., Tianingrum N.A., Safaah N. (2024). A Systematic Review of Determinants Influencing Family Planning and Contraceptive Use. Iran. J. Nurs. Midwifery Res..

[33] Rutstein S.O., Johnson K. (2004). The DHS Wealth Index.

[34] Cleland J., Shah I.H., Daniele M. (2015). Interventions to Improve Postpartum Family Planning in Low- and Middle-Income Countries: Program Implications and Research Priorities. Stud. Fam. Plan..

[35] Demissie G.D., Akalu Y., Gelagay A.A., Alemnew W., Yeshaw Y. (2022). Factors associated with decision-making power of married women to use family planning in sub-Saharan Africa: A multilevel analysis of demographic health surveys. BMC Public Health.

[36] Withers M., Dworkin S.L., Onono M., Oyier B., Cohen C.R., Bukusi E.A., Newmann S.J. (2015). Men’s Perspectives on Their Role in Family Planning in Nyanza Province, Kenya. Stud. Fam. Plan..

[37] Sitrin D., Jima G.H., Pfitzer A., Wondimu C., Belete T.W., Pleah T., Assefa B., Kebede T., Regassa E., Usman E.A. (2020). Effect of integrating postpartum family planning into the health extension program in Ethiopia on postpartum adoption of modern contraception. J. Glob. Health Rep..

[38] Widyastuti Y., Akhyar M., Setyowati R., Mulyani S. (2023). Relationship Between Gender Equality and Husband Support in the Use of Postpartum Family Planning (PPFP). SAGE Open Nurs..

[39] Chandrasekar A., Warren E., Free C., Mbogua J., Curtin E., Gazeley U., Wong G., Church K., McCarthy O. (2024). mHealth interventions for postpartum family planning in LMICs: A realist review. PLoS Glob. Public Health.

[40] Zulu E.M., Sukwa T. (2020). Impact of mHealth on contraceptive use among women and men of reproductive age in low- and middle-income countries: A systematic review and meta-analysis. Trop. Med. Int. Health.

[41] Iftikar T., Rauf R., Qamar S., Khaliq N., Ejaz S., Faisal J. (2024). The Role of Husbands’ Awareness in Enhancing Postpartum Maternal Health in Rural Islamabad: A Cross-Sectional Analysis. J. Soc. Obstet. Gynaecol. Pak..

[42] D’Exelle B., Ringdal C. (2022). Women’s use of family planning services: An experiment on the husband’s involvement. J. Dev. Econ..

[43] Lutkenhaus R.O., Jansz J., Bouman M.P. (2019). Tailoring in the digital era: Stimulating dialogues on health topics in collaboration with social media influencers. Digit. Health.

[44] Konkor I., Sano Y., Antabe R., Kansanga M., Luginaah I. (2019). Exposure to mass media family planning messages among post-delivery women in Nigeria: Testing the structural influence model of health communication. Eur. J. Contracept. Reprod. Health Care.

[45] Rogers D., Snyder L.B., Rego M. (2021). The Impact ofMass Media-Delivered Family Planning Campaigns in Low- and Middle-Income Countries: A Meta-Analysis ofAdvertising and Entertainment-Education Format Effects. Stud. Fam. Plan..

[46] Hill J., McGinn J., Cairns J., Free C., Smith C. (2020). A Mobile Phone-Based Support Intervention to Increase use of Postabortion Family Planning in Cambodia: Cost-Effectiveness Evaluation. JMIR mHealth uHealth.

[47] UNFPA (2020). Costing the Three Transformative Results.

