# Supplementary material for: Effect of mHealth on Postpartum Family Planning and Its Associated Factors Among Women in South Ethiopia: A Cluster-Randomized Controlled Trial
Source: J Clin Med. 2025 Dec 9;14(24):8703. doi: 10.3390/jcm14248703 (PMC12733925; doi:10.3390/jcm14248703)
Supplement: Supplementary file 1 [file jcm-14-08703-s001.zip › jcm-4008774-supplementary.pdf]

Supplementary Material: mHealth SMS System Implementation and Monitoring Framework

Intervention schedule

1. Overview of SMS Delivery Framework

The mHealth intervention was designed to deliver structured health messages and reminders to enrolled mothers via SMS. The system was implemented using FrontlineSMS, hosted securely at the Arba Minch University Health Informatics Department, and operated through a Virtual Private Network (VPN) provided by Ethio-Telecom. All messages were transmitted in Amharic, tailored to the gestational or postpartum stage, and limited to ≤160 characters for clarity and readability.

Parameter	Description
SMS Platform	FrontlineSMS (open-source health messaging system)
Server Hosting	Arba Minch University, Health Informatics Department
Transmission Provider	Ethiopian Telecommunication Corporation (Ethio-Telecom)
Language	Amharic
Average Message Length	≤160 characters
Delivery Frequency	Once per week (information message + reminder)
Delivery Mode	Automated, secure via VPN
Monitoring Interval	Weekly (system logs + manual verification)
Preferable time	7:00 PM and 8-10:00 AM if the mother has not opened the message

2. Message Categories and Content Themes

**Category**	**Example Message**	**Timing**	**Behavioral Model Basis**
**ANC Reminder**	"Your next ANC visit is this week. Early visits keep you and your baby healthy."	Every two weeks until birth	Cue to Action (HBM)
**Prelacteal feeding**	"Avoid giving water, butter, or other foods before breastfeeding — these can cause illness and reduce your baby's milk intake."	Every two weeks (until birth)	Perceived Susceptibility & Perceived Severity (HBM)
**Postpartum FP Information**	"Plan your next pregnancy. Discuss family planning with your health worker."	Every two weeks to 0–6 months postpartum	Attitude & Intention (TPB)
**Exclusive Breastfeeding**	"Feed your baby only breast milk for six months — it's all they need."	Every two weeks to 0–6 months postpartum	Perceived Benefit (HBM)
**Partner Involvement**	"Talk to your partner about birth spacing. Family planning is a joint decision."	Throughout	Subjective Norm (TPB)
**Vaccination Reminder**	"Your child's vaccination is due this week. Visit your nearest health post."	As per the EPI schedule	Cue to Action (HBM)

3. Message Monitoring and Quality Assurance

Message delivery was monitored from dispatch through read-status confirmation, with subsequent verification of engagement as required.

Indicator	Definition	Monitoring Tool	Reporting Frequency
**Message Sent**	Total number of SMS successfully queued in the system	FrontlineSMS dashboard	Weekly
**Delivery Success Rate**	Percentage of messages delivered to recipients' phones	SMS Delivery Report	Weekly
**Read Confirmation Rate**	Percentage of delivered messages confirmed as opened/read	Delivery log + follow-up call (sample)	Monthly
**Non-delivery Rate**	Percentage of messages not delivered due to network failure or wrong number	Delivery log	Weekly
**Loss to Follow-Up**	Percentage of participants unreachable after ≥3 contact attempts	Field verification sheet	Every three months
**Opt-out Rate**	Percentage of participants who declined further messaging	Opt-out log	Continuous
**System Downtime**	Duration of any system interruptions or server failures	Server Log	Ad hoc

4. Non-Delivery and Loss-to-Follow-Up Management

Scenario	Response Strategy	Responsible Person
**Non-delivery (network failure)**	Verify participant phone status and resend within 24 hours.	Technical Assistant & PI
**Phone switched off >3 days**	Attempt contact through alternate number	Principal investigator (PI)
**Participant relocated**	Update record in FrontlineSMS, classify as LTFU.	Data Manager & PI
**Repeated unread messages (≥3 consecutive)**	Conduct a phone check-in to confirm receipt or continued consent.	Field Enumerator & PI
**Participant withdrawal**	Document opt-out in the log and stop message delivery.	Data Manager & PI

5. Data Integrity and Confidentiality

- All data (SMS logs, read receipts, and follow-up records) were stored on secure, password-protected university servers.
- Only authorized researchers accessed system logs.
- Participants' phone numbers were anonymized using coded identifiers before analysis.
- All procedures adhered to AMU-IRB ethical approval (Ref. No. 1326/2022) and Declaration of Helsinki guidelines.
- Four participants were available for the baseline data collection but were not available during commencing the trial. They are not accessible and are not established in the trial.

6. Metrics

1. **Participants**

During the assessment of the first intervention objective (prelacteal feeding), four participants were lost to follow-up (340-4 = 336; 336/340*100 = 99%). And across the next objectives, overall, 5 women were lost to follow-up (340-5 = 335; 335/340*100 = 98.5%). Generally, during the two assessment periods, the overall loss to follow-up was approximately 1%. i.e., 99% of participants stayed in the study.

2. **Delivery Success Rate**

Overall, 12,240 messages (both reminders and information) were planned, and 12,168 (99%) were achieved. The FrontlineSMS software auto-generates the Delivery Report after each batch.

- In the first part of the trial (third trimester to assessment of prelacteal feeding five months), 5,408 messages were sent.
- In the second part of the trial (birth to sixth month), 6760 messages were sent.

$$5{,}408 + 6760 = 12{,}168;\ 12{,}168/12{,}240 \approx 99\%$$

3. **Read / Open Confirmation**

Reading and opening the message status was verified through system log (SMS) assessment and Self-report via follow-up telephone survey.

- Randomly sampled 100 participants and asked if they read the message
- An average of 95 out of 100 women reported reading the message from three verification assessments (93 +95+98)/3 = 95 self-reported

4. **Non-delivery**

Non-Delivery Rate = 100−Delivery Success Rate

$$100-95 = 5\%$$

5. **Loss to Follow-Up (LTFU)**

Five women were LTFU from the follow-up tracking log.

LTFU= 5/680*100 = 0.73%

During recruitment, 680 women were selected. However, at the baseline data collection stage, four mothers were not included. Two were unreachable with no available evidence, one declined participation because her husband did not permit her to receive messages, and another reported that she was moving to live in Addis Ababa and, therefore unable to consent. Thus, the baseline comparison between included and excluded participants is limited to a few variables, specifically the mother's age and gestational age. During an additional one mother drew auto of the study due to the loss of the baby. This was just during the data collection of the first part of the study. So, she ceased the follow-up.

Included vs excluded

The mean age of women lost to follow-up (LTFU) was 25.4 ± 1.91 years, and their mean gestational age was 23.6 ± 0.98 weeks. By contrast, the mean age of included mothers was higher (27.6 ± 0.20 years), while their mean gestational age was slightly lower (22.88 ± 0.09 weeks). Given the characteristics of the LTFU sample, the nature of the intervention, and its potential influence on participants, it cannot be concluded that the observed missingness was related to the intervention itself.

7. Common Reasons for Withdrawal or Loss to Follow-up

**Category**	**Examples**	**Typical Implication**
**Participant-related**	Relocation, lack of interest, withdrawal of consent	May introduce bias if related to exposure or outcome
**Health-related**	Adverse events, disease progression, death	Can bias results if associated with treatment
**Study-related**	Protocol violations, lost contact, and administrative error	Usually random, but must be documented
**Other/Unknown**	Unspecified dropout	A high rate may signal data quality issues

Overall, the study has low loss to follow-up, not associated with any sensitive outcome. One woman withdrew from the study, reporting fear of a suspicious husband. However, this had happened before the trial commenced. There is no evidence that any of the LTFU are associated with treatment.

8. Baseline Comparison Table

**Characteristic**	**Included (n = 676)**	**Excluded (n = 4)**	**p-value**
Age (years), mean ± SD	27.6 ± 0.20	25.4 ± 1.91	0.7
Gestational age (mean ± SD)	12.4 ± 2.1	12.1 ± 2.3	0.26

Opt-Out or Withdrawals

One woman withdrew from the trial. This was manually confirmed after making a call to the mother. She reported pressure from her husband.

Withdrawal = 1/340*100 = 0.3%

NB: One of the mothers reported loss of the baby to respiratory illness in the second month after birth. This follows a similar calculation to the withdrawal above.

9. SMS Engagement Statistics (Trial Summary)

Metric	Value	Interpretation
Total messages sent	12,240	Represents 340 participants in the intervention arm
Delivery success rate	99%	Excellent technical performance
Read/open confirmation	95% (self-reported subset)	High participant engagement
Non-delivery (network failure)	5%	Minimal technical loss
Loss to follow-up	5 participants (≈0.73%)	Strong retention
Opt-out/withdrawal	0.3%	Low attrition, indicating acceptability
Death of child	0.3%	No comment

10. Implementation Supervision

Weekly supervision was conducted by the **PI** and **Technical Assistant**, with monthly reporting to the Principal Investigator. Midline reviews were held to evaluate message timing, participant engagement, and data consistency.

11. Lessons Learned

- Continuous system monitoring ensures near-perfect delivery rates.
- Including human feedback loops (phone check-ins) minimizes participant dropout.

- Behavioral model–guided messages improve engagement and perceived usefulness.
- Integration with local health system workflows enhances sustainability and ownership.

12. Recommended Template for National mHealth Scale-Up

**Function**	**Responsible Entity**	**Tool/Platform**	**Frequency**	**Remark**
Message scheduling	Regional Health Bureau ICT Desk	FrontlineSMS / RapidPro	Weekly	Local HF
Monitoring & troubleshooting	MOH Digital Health Unit	National SMS Log Dashboard	Weekly	Local HF
Ethical oversight	IRB / Health Research Directorate	Compliance Tracker	Biannual	Local HF
Data reporting	MOH RMNCH Directorate	DHIS2	Quarterly	Local HF
Privacy & confidentiality	Ethio-telecom &MOH	Server/network	Continuous	Local HF
Support	Universities	Training/research	Quarterly	Local HF

NB: Local Health Facility (HF) is responsible for uploading and following messages, while other bodies control the system. All activities will be done with knowledge of local HFs.
